# The role of women’s resources in the prediction of intimate partner violence revictimization by the same or different aggressors

**DOI:** 10.3389/fpsyg.2022.1014683

**Published:** 2022-09-28

**Authors:** Ana Bellot, María Izal, Ignacio Montorio

**Affiliations:** Department of Biological and Health Psychology, Autonomous University of Madrid, Madrid, Spain

**Keywords:** intimate partner violence, revictimization, women, emotional resources, social resources, material resources

## Abstract

The literature studying the characteristics associated with revictimization in Intimate Partner Violence (IPV) is heterogeneous and inconclusive. The absence of studies on the role of the emotional variables of the victims and the failure to distinguish revictimization by the same or different aggressors are two of the main limitations in this area of research. The aim of this work was to study the relative contribution of the material, social, and emotional resources available to IPV victims in predicting revictimization by the same or different perpetrators. The sample consisted of 290 women registered in the city of Madrid who had filed at least one police report for intimate partner violence. The material resources of the victims were evaluated through their level of monthly income and employability status, the social resources through perceived social support, and the emotional resources through emotional regulation and coping strategies. Hierarchical multinomial logistic regression models were estimated to predict single-offender victimization (SRV), same-offender revictimization (VSRSA), and multiple-offender revictimization (VSRDA). The results revealed that: (1) differentiating between revictimization by the same and different aggressors improved the fit of the model by 50.8% compared to when only differentiating between victimized and revictimized women; (2) material resources had no significant weight in the prediction of any type of revictimization; (3) SRV women had more social support than VSRDA women (ExpB = 1.027; *p* < 0.011); (4), those victims who had made several reports to the authorities of violence by different aggressors (VSRDA), had worse emotional regulation than those victims who had made a single report to the authorities (VSRs; ExpB = 2.934; *p* < 0.026); and (5) VSRDA obtained the worst mental health indexes and they used more coping strategies based on positive reappraisal than the VSR women (ExpB = 0.863; *p* < 0.009) and those victims with several reports by the same aggressor (VSRSA; ExpB = 0.891; *p* < 0.028). These results show that being a victim of several episodes of intimate partner violence by different aggressors should be understood as a form of revictimization of great severity associated with worse emotional regulation and less social support.

## Introduction

Intimate partner violence (IPV) is a complex phenomenon that has become a major public health problem [World Health Organization (WHO), 2013]. Women victims of IPV often experience psychological consequences, such as post-traumatic stress disorder (PTSD), depression, anxiety, substance use disorders, eating disorders, somatic complaints, and suicidal tendencies (Iverson et al., 2013). In addition, having suffered violence on one occasion increases the risk of being assaulted again by the same partner and also in future relationships. Specifically, between 22.9 and 56% of women who experience gender-based violence had already had previous histories of victimization in previous intimate partner relationships (Ørke et al., 2021). It has been shown that the consequences of re-experiencing abusive situations at the hands of a partner or ex-partner are more severe and long-lasting than when there is a single episode of violence, which more negatively affects the victim’s ability to recover psychologically and emotionally (Kuijpers et al., 2011; Iverson et al., 2013).

Most studies on revictimization focus on analyzing the variables associated with recidivism in perpetrators and have tended to ignore the factors associated with the victims for fear of blaming them (Orke et al., 2018). However, knowing which strategies and resources are associated with revictimization by the same or different partners could help to enhance a more active and effective coping mechanism with situations of violence, without, in any case, discharging the aggressor of his responsibility for the violent behavior.

In this sense, the normative resource theory states that, in couple relationships, people with greater access to material, social, and emotional resources will be able to exert greater positive control over their partners (Crosbie-Burnett and Giles-Sims, 1991). From this point of view, women who are more economically, emotionally, and socially dependent on their partners will be more likely to be revictimized, while those who have more of the three types of resources will be in a more favorable situation to avoid revictimization by the same or different partners (Goodman et al., 2005).

This theory has been empirically contrasted in numerous studies with respect to material resources. Thus, socioeconomic status has been shown to be a strong protective factor in IPV revictimization (Coolidge and Anderson, 2002; Hirschel and Hutchison, 2003; Caetano et al., 2005; Person, 2018). However, employability although less studied and with less clear results, also seems to have a mainly protective effect against revictimization (Crandall et al., 2004; Hayes, 2018; Ørke et al., 2021). Social or interpersonal resources, mainly assessed through the perceived social support of IPV victims, have also frequently been included among the predictors associated with revictimization in gender-based violence and in most studies they are given a mainly protective role (Goodman et al., 2005; Valentine et al., 2016; Ogbe et al., 2020; Ørke et al., 2020). Finally, with respect to emotional resources, there is a line of research which has focused on exploring the victims’ use of both emotional regulation and coping strategies in the face of stress to mitigate the consequences of IPV (Crowe and Murray, 2015; Puente-Martinez et al., 2021). Even so, the evidence that has analyzed emotional regulation and coping processes in the population of IPV victims is scarce and heterogeneous when compared with the high number of studies conducted to examine the role of material and interpersonal resources (Keeling et al., 2016; Orke et al., 2018).

The study of coping strategies has classically followed an inter-individual approach that involves analyzing the different strategies grouped into the coping styles that the individual may use across different situations and stressors (Lazarus and Folkman, 1984; Cohen, 1987; Waldrop and Resick, 2004). From this approach, “engagement” vs. “disengagement” coping styles are differentiated (Lewis et al., 2006). The first is usually defined as proactive ways of coping with problems, it is associated with a healthy style of coping with stress and usually includes strategies, such as focusing on the problem, cognitive reappraisal, or emotional expression (Iverson et al., 2013). The second, on the contrary, refers to more passive coping strategies, it is negatively associated with healthy stress coping styles and usually includes avoidance strategies, negative self-focus or delusional thinking (Sandín and Chorot, 2003; Iverson et al., 2013). When transferred to the field of IPV, it has been recommended that women victims be trained in the use of “engagement” strategies which would provide them with a protective role against revictimization, while passive strategies would favor revictimization (Iverson et al., 2013; Puente-Martinez et al., 2021).

In contrast to the previous approach, it is currently considered more appropriate to analyze each coping strategy separately, examining their functionality in a given context and population, rather than grouping them into watertight and general categories that underestimate the complexity of the reality experienced by women victims of IPV (Lewis et al., 2006; Sullivan et al., 2010; Puente-Martinez et al., 2021). A review on coping in female victims of domestic violence supports the use of an intra-individual approach in the study of coping because coping tends to vary as a function of the characteristics of the stressful situation (Waldrop and Resick, 2004).

In accordance with the above, it is considered that the situational and personal variables that model the functionality of the different coping strategies have a short trajectory of study (Meyer et al., 2010). It seems that the level of perceived control over the situation, as well as the contextual factors that determine the level of change in the situation, are factors which have a great impact on the choice of the coping strategy used and its functionality (Kacot, 2003; Puente-Martinez et al., 2021). In women victims of IPV, the cognitive avoidance strategy, understood as continuous attempts to keep the mind busy, spending time away from home, or distracting themselves with another activity, is frequently encountered, arguing that women resort to it in the face of a lack of controllability over the violent situation (Walsh et al., 2011; Pérez-Tarrés et al., 2017; Ozturk et al., 2019). However, the use of this strategy has been shown to be moderated by other contextual factors such as the duration and frequency of violence (Waldrop and Resick, 2004; Pérez-Tarrés et al., 2017). Specifically, it has been observed that at the beginning of the relationship women use more problem-focused strategies aimed at dialoguing, confronting, or understanding the situation (Miracco et al., 2010; Pérez-Tarrés et al., 2017). In the absence of contingency between their behavior and the presence or not of violence, the locus of control becomes increasingly external to the victim, corresponding with an increase in the use of avoidance strategies to reduce any type of confrontation. As the relationship progresses and the abuse continues, conformist attitudes and feelings of guilt and hopelessness increase, which are also associated with a greater presence of PTSD symptomatology in IPV (Iverson et al., 2013; Pérez-Tarrés et al., 2017). At this point, some authors claim that coping strategies based on a positive reappraisal of the situation become a key for the adaptation to the situation of violence. Thus, Zink et al. (2006) state that when faced with a situation of total uncontrollability, “women change the concept they have of themselves, their partners and their relationships to learn to live with the abuse” (p. 644). This evolution of coping strategies as the relationship progresses fits with learning model of Walker (1979) to explain what the use of different coping strategies in IPV depends on. According to this author, the choice of certain strategies in the present is the result of those behaviors being useful in the past, so that women who use strategies that prove consistently ineffective over an extended period of time develop a state of learned helplessness in which even escape reactions are blocked. Unfortunately, these results have only been analyzed in a limited number of studies which have mainly been descriptive and qualitative.

From this point of view, the functionality of coping strategies is completely linked to contextual and personal factors and points to the limited usefulness of predetermined divisions of coping strategies into global dimensions of engagement and disengagement. Given that abusive relationships involve specific circumstances in which the use of strategies *a priori* considered “healthy” may be a risk factor in the maintenance of violence (Goodman et al., 2005), it is likely that the use of an intra-situational approach that takes into account the personal and situational characteristics of the victim is the most appropriate way to study the role of coping strategies in the field of IPV revictimization.

Regarding emotional regulation, which is the second emotional resource considered of interest in the field of IPV study, the evidence for this is much more limited than for coping strategies (Muñoz-Rivas et al., 2021). The scarce literature available shows that women with IPV experiences have greater difficulties in accepting and acknowledging their emotions (Ullman et al., 2009) and that revictimization is associated with weaker emotional regulation strategies (Classen et al., 2005). More specifically (Muñoz-Rivas et al., 2021) found that there was an interaction effect between the type of revictimization suffered, either by the same aggressor or by different aggressors, and emotional regulation in predicting PTSD symptomatology in women victims of IPV. Thus, in the group of women revictimized by multiple aggressors, the difference in PTSD symptomatology between the group with good and poor emotional regulation was significantly different from the differences found in the groups of women revictimized by the same aggressor, and those who had not been revictimized. These results are congruent with the multiple studies that in the last decade have presented emotional regulation as a determinant factor in the maintenance of multiple clinical problems (Gross, 2015; Seligowski et al., 2015; McRae and Gross, 2020) and, moreover, point out that its role in IPV may be especially important when violence is more frequent and sustained over time.

### Current study

In this paper, the different types of resources are studied together to examine the relative contributions of each in predicting different forms of revictimization in IPV. A precedent for the present study can be found in Goodman et al. (2005) who provided empirical support for normative resource theory by analyzing the predictive role in revictimization of each type of resource in the presence of the others. To assess the victims’ material resources, they used socioeconomic status and employability, for social or interpersonal resources they measured social support, and for emotional resources they assessed quality of life and passive and confrontational coping strategies in the face of violence. In their results they found that only social support was a protective factor for revictimization and that confrontation strategies functioned as a risk factor, whilst the other predictors of the model were insignificant.

The present study is similar to that of Goodman et al. (2005) with respect to how material and social resources are considered, although it differs notably in the way in which emotional resources are considered, as well as in the way in which revictimization is assessed. Firstly, emotional resources are assessed through the level of emotional regulation and through coping strategies using standardized scales. Emotional regulation is considered to be a construct that more accurately and concretely represents the emotional resources of victims than their quality of life, which can be understood more as an outcome. Similarly to Muñoz-Rivas et al. (2021), the aim is to test whether belonging to more or less efficient emotional regulation groups discriminates in the risk of revictimization. Regarding coping strategies, following an intra-personal approach, the aim is to avoid resorting to the grouping of coping strategies into generic categories, i.e., engament vs. disengament, and to evaluate the role of some of the main coping strategies related to IPV separately. Attending to the importance of considering the controllability, duration, and frequency of violence for the analysis of the functionality of coping strategies, the aim is to check whether, depending on the type of revictimization (developed below): (1) avoidance strategies are still the most used, (2) whether the increase in feelings of hopelessness, guilt, and learned helplessness means that women tend to focus on negative aspects (Pérez-Tarrés et al., 2017) or, if on the contrary, following the findings of Zink et al. (2006) women tend to positively re-evaluate the situation as a coping strategy in the face of the perceived impossibility of escape from violence.

Secondly, this study will take into account whether revictimization occurs at the hands of the same or different aggressors, as this has been shown to be a relevant moderating variable. Those women revictimized by multiple aggressors are more likely to have greater clinical symptomatology, to have suffered abuse in childhood, to have an avoidant attachment pattern and to have problems of addiction to illegal drugs (Kuijpers et al., 2011; Orke et al., 2018). Moreover, attending to the importance of context in assessing the role of emotional resources, this differentiation may give greater precision to the interpretation of the results.

The objectives are as follows:

- To test the role of material, social, and emotional resources in the prediction of revictimization. It is hypothesized that emotional resources, despite their lower representation in the literature, will have a similar or greater weight than social and material resources.
- To incorporate emotional variables in the analysis of the results. It is hypothesized that emotional regulation will play an important role in the study of revictimization as it does in many other fields.
- To test the role of different types of revictimization. It is hypothesized that revictimization by different aggressors will lead to a higher level of vulnerability in the victims.
- To analyze the role of specific coping strategies in the context of different types of revictimization. Specifically, the strategies of avoidance, positive reappraisal, and negative self-focus will be evaluated and it is hypothesized that having suffered revictimization at the hands of the same or different aggressors will influence the type of specific coping strategy employed by the victim.

## Materials and methods

### Participants

The study sample consisted of 290 women victims of IPV who were included in the Sistema de Seguimiento Integral de los casos de violencia de género (VioGen) of the Spanish Ministry of the Interior belonging to the Community of Madrid since 2014. Forty-eight women were excluded from the analysis for reasons explained in the procedure section, which reduced the final sample size to 242 participants.

### Procedure

This study was developed in collaboration with the Secretary of State for Security of the Spanish Ministry of the Interior, which is responsible for the VioGén System that registers any complaint made by a woman regarding physical, sexual, or psychological IPV at the national level. For each complaint, the victim’s data are recorded and information is attached regarding her socioeconomic context, history of victimization and the corresponding legal and judicial assessment.

For research purposes, the participants in the study were selected according to the following inclusion criteria: (a) being an active case after filing a complaint within the VioGén System because of physical, sexual, or psychological IPV; (b) having a court sentence for police protection measures; and (c) being of legal age. In addition, in order to have all the profiles of victims of IPV represented according to the number of times they have filed a complaint and the multiplicity of aggressors the following were selected:

- Victims of a single episode of gender-based violence (*victims single report, VSR*): women victims included in VioGen since 2014 who, after a single report, have not filed any subsequent report. Those women who were interviewed as victims of a single episode of gender-based violence, but in the interview stated that they had suffered other episodes of violence from the same or different partners that they did not report were excluded from the analyses.
- Victims of several episodes of gender-based violence perpetrated by the same partner (*victims with several reports by the same aggressor*, *VSRSA*): female victims included in VioGen since 2014 who have filed several reports by the same aggressor.
- Victims of multiple episodes of gender-based violence perpetrated by multiple partners (*victims with several reports by different aggressors, VSRDA*) female victims included in VioGen since 2014 who have filed multiple reports by different aggressors.

Once the extraction was performed, the police officers were asked to inform the victims about the possibility of collaborating in the study. The women who agreed to participate were contacted by telephone to obtain their voluntary consent to participate. The interviews lasted approximately 3 h and were conducted at the place of preference of the participants, usually their home. After signing the informed consent form, the interview was structured and guided by a standard evaluation protocol. The procedure was approved by the Research Ethics Committee of the Universidad Autónoma de Madrid (CEI-941720).

### Instruments

- An *ad-hoc* questionnaire was developed to collect psychosocial information on the victims: age, nationality, marital status, educational level, income level, employability, and time elapsed since the complaint was filed and the victim’s estimated probability of being assaulted again by another partner in the future.
- Emotional Processing Scale (EPS-25; Baker et al., 2010) consists of 25 items organized into five factors (suppression, avoidance, unregulated emotion, impoverished emotional experience, and signs of unprocessed emotions) that allow the calculation of an overall score. Each item is rated on a 10-point Likert-type scale (0 “completely disagree” to 9 “completely agree”). The range of possible total scores was from 0 to 225, with a higher score indicating lower emotional regulation ability. In the present study, the reliability estimated by Cronbach’s alpha coefficient was 0.954 [95% CI = 0.945–0.963] and the same value was used for the Omega coefficient. In the subscales, the values ranged between 0.79 and 0.896 for Cronbach’s alpha coefficient and between 0.809 and 0.872 for the Omega.
- Post Traumatic Stress Disorder (PTSD) Revised Symptom Severity Scale (EGS-R) is a modified and updated version of the EGS used to assess the severity of PTSD symptoms based on the DSM-5 diagnostic criteria (Echeburúa et al., 2016). It consists of 21 items rated on a four-point Likert-type scale (0 “Not at all” to 3 “Five or more times per week/much”). It analyzes the factors reexperiencing, behavioral/cognitive avoidance, cognitive alterations and negative mood, and increased physiological reactivity. The higher scores equate to a greater severity of symptomatology and in this study it was used, together with the DASS, to assess the level of adjustment of the different types of victim. With the present sample, the reliability estimated by Cronbach’s alpha coefficient was 0.909 [95% CI = 0.89–0.925] and 0.908 on the Omega coefficient for the total scale. In the individual scales, the alpha coefficients took values between 0.747 and 0.883 and the Omega coefficients between 0.827 and 0.869.
- An abbreviated version of the Depression, Anxiety, and Stress Scales (DASS-21). Specifically, the Spanish version validated by Antúnez and Vinet (2012) was used. The DASS-21 assesses in a self-reported manner the presence and intensity of the affective states of depression, anxiety and stress in the latter. It has a total of 21 items, with four response alternatives in a Likert format, ranging from 0 (“It does not describe anything that happened to me or I felt during the week”) to 3 (“Yes, this happened to me a lot, or almost always”). Each subscale has seven items and its total score is calculated with the sum of the items belonging to that subscale, ranging from 0 to 21 points. The Depression scale assesses dysphoria, meaninglessness, self-criticism, lack of interest, and anhedonia. The Anxiety scale considers subjective and somatic symptoms of fear, autonomic activation, situational anxiety, and subjective experience of anxious affect. The Stress scale assesses persistent non-specific activation, difficulty relaxing, irritability, and impatience. Cronbach’s alpha for the total scale in the present sample was 0.965 [CI: 0.958–971]. In the depression scale, the value obtained was 0.924 [CI: 907–938], 0.902 [CI: 882–920] for the anxiety scale, and 0.908 [CI: 0.888–0.925] in the stress scale. The Omega coefficients were 0.966, 0.926, 0.907, and 0.910, respectively.
- Multidimensional Scale of Perceived Social Support (EMAS) adapted to Spanish by Ruiz Jiménez et al. (2017). This is a 12-item instrument that collects the levels of social support perceived by friends, family, and relevant people to whom it is administered. Each item has seven alternatives, where value 1 means “Strongly disagree” and value 7 “Strongly agree,” so that the total score on the scale ranges from 12 to 84 points. The higher scores indicate greater perceived social support. In the total scale, Cronbach’s alpha was 0.918 [CI: 901–933] and the Omega coefficient took a value of 0.891. In the three subscales, values between 0.842 and 955 were obtained for Cronbach’s alpha and between 0.847 and 0.954 for the Omega coefficient.
- Stress Coping Questionnaire (CAE) validated in Spanish by Sandín and Chorot (2003). The theoretical justification provided has led to the selection of the avoidance scale for this study, as it is the most used coping strategy in IPV (Ozturk et al., 2019); the negative self-focus scale, for encompassing negative coping dimensions, such as guilt, hopelessness, and resignation associated with the maintenance of violent situations (Pérez-Tarrés et al., 2017); and the positive reappraisal scale, for its key role in the face of learned helplessness developed in revictimized women (Zink et al., 2006). Each subscale is made up of six items that participants evaluate on a scale of 0–4 according to the frequency with which they made use of the coping strategy raised to deal with the violence. Some examples of items from the avoidance scale are: (1) “When the problem came to my mind, I tried to concentrate on other things,” (2) “I turned to work or another activity to forget about the problem,” and (3) “I went out to the movies, to dinner, for a walk, etc., to forget about the problem.” In the case of the positive reappraisal scale, some examples of its items are: (1) “I tried to focus on the positive aspects of the problem,” (2) “I understood that things other than the problem were important to me,” and (3) “I personally experienced that every cloud has a silver lining.” Finally, the negative self-focus scale is made up of items such as: (1) “I convinced myself that no matter what I did, things would always go wrong” (2) “I did nothing concrete since things are usually bad,” (3) “I understood that I was the main cause of the problem,” and (4) “I felt helpless and unable to do anything positive to change the situation.” Reliability analyses estimated a Cronbach’s alpha coefficient of 0.66 [CI: 0.59–0.724] for the avoidance scale, a value of 0.754 [CI: 0.70–0.801] for the negative self-focus scale, and 0.63 [CI: 0.55–0.701] on the positive reappraisal scale. The Omega coefficients were 0.657, 0.749, and 0.621, respectively. The reliability coefficients obtained are somewhat low, but acceptable and consistent with those obtained in the original validation article, in which the alpha coefficients for the three scales ranged from 0.64 to 0.76 (Sandín and Chorot, 2003).

### Statistical analysis

Initially, the internal consistency of the scales used in the study was analyzed using the Alpha and Omega coefficients. Coefficients above 0.60 were considered acceptable (Taber, 2018). Descriptive analyses were performed for sociodemographic variables and for the different types of resources, and one-factor ANOVAs were performed to contrast whether there were differences in means between the three types of victims in the resources assessed and in their clinical adjustment. Hierarchical binary logistic regression models were estimated to determine the weight of each resource in predicting whether or not women had been revictimized. The types of resources were entered in blocks using their recognition in previous literature as a criterion: firstly, material resources, secondly, social resources, and lastly, emotional resources. The final model was carried out by means of a hierarchical multinomial regression analysis which, with respect to the binary logistic regression analysis, allows prediction of category membership of a polytomous variable with more than two categories. This model was used to predict membership in the VSR, VSRSA, and VSRDA groups on the basis of the resources evaluated. To facilitate the interpretability of the results, taking (Muñoz-Rivas et al., 2021) as a reference, in the regression models, the variable emotional regulation was dichotomized forming two groups divided by the median that differentiated between women with high and low emotional regulation. The means between the two groups differed significantly (T = −25.98; *p* < 0.0001; 95% CI of difference = −4.436 to −3.811).

In order to be able to affirm that the multinomial regression models work correctly, the assumptions of independence of the predictor variables, collinearity, dispersion proportional to the mean, and linearity between the independent variables were tested (Pardo and Ruiz, 2012). In this study, no assumption was violated. The assumption of independence did not need to be checked because, as this was a retrospective study, there was no sequential data collection. Regarding the diagnoses of collinearity, high tolerance levels (between 0.738 and 0.880) and low variance inflation factors (between 1.13 and 1.36) were obtained for all variables, which ruled out problems due to excessive collinearity between predictors. The scale parameter to evaluate the proportional dispersion of the mean was 1.09, which indicates the absence of overdispersion and underdispersion, being a value very close to 1. Finally, polynomial analyses were performed to evaluate what type of trend relationship existed between the logit of the dependent variable and the independent variables. For all predictors, only the linear trend was significant, except for the variables positive reappraisal and avoidance strategies, where neither the linear nor the quadratic trend was significant, so that the null hypothesis of non-linearity could not be rejected.

## Results

Descriptive analysis showed that the mean age of the women was 37.88 years, 65.8% were of Spanish origin, 58.9% of the participants had a high school or university education, 27.8% had vocational training, 12.9% had primary education, and less than 1% had no education. A total of 33.5% were women victimized on a single occasion, 41.7% were victimized on more than one occasion by the same aggressor, and 24.8% were victimized more than once by different aggressors.

Analyses to assess whether the sociodemographic variables age, level of education, and time elapsed since the complaint and the expectation of being victimized again differed among the three types of victims showed statistically insignificant relationships, except for the variable that recorded whether the victim considered that she would probably or surely be assaulted again by another partner in the future. Differences were found between the group of women revictimized by multiple aggressors (VSRDA) and the group of women victimized on one occasion (VSR) and revictimized by the same aggressor (VSRSA). Specifically, 39.7% of the VSRDA women answered that they would probably/surely be assaulted again compared to 22.4% of the VSRSA women (*p* < 0.042) and 17.5% of the VSR (*p* < 0.008). Table 1 shows descriptive statistics for all variables assessed.

**Table 1:** Descriptive statistics.

	Average/%	Deviation
Age	37.88	10.671
Level of education		
No education	0.4%	
Primary	12.9%	
Vocational Training	27.8%	
Baccalaureate/University	58.9%	
Nationality		
Spanish	65.8%	
Rest	35.2%	
High self-reported likelihood of revictimization by another offender		
VSR	17.5%	
VRSA	22.4%	
VSRDA	39.7%	
Monthly income		
Up to 450 €.		
From 451 to 600 €	13.6%	
From 601 to 1,000 €	25.6%	
From 1,001 to 1,500 €.		
From 1,501 to 2000 €	6.6%	
From 2001 to 3,000 €.	4.1%	
More than 3,000 €.	1.2%	
Employability		
Works full time/part time	64.9	
Does not work	35.1	
Positive CAE reassessment	14.9312	4.78572
CAE avoidance	13.8095	4.90502
Negative self-focusing CAE	13.6244	5.61323
Emotional regulation EPS	3.5851	2.40153
Global_Support	66.1342	17.29826

We analyzed whether there was a relationship between the different types of resources and sociodemographic variables. It was found that educational level was positively associated with income level (*r* = 0.390; *p* < 0.01) and with social support (*r* = 0.144; *p* < 0.05), and positive reappraisal strategies were negatively associated with victim’s age (*r* = −0.190; *p* < 0.001).

Analyses to assess the clinical adjustment of the different types of victims showed that VSRDAs differed significantly from VSRs and VSRSAs in post-traumatic symptomatology as well as in the stress, anxiety, and depression scales. Thus, they presented a higher level of PTSD, anxiety, stress, and depression symptomatology than the other two groups. In contrast, no differences were found between the VSR and VSRSA groups for any of the scales (Table 2).

**Table 2:** Clinical setting by victim group.

Dependent variable	(I) tipovictima	(J) tipovictima	Difference of means (I-J)	Sig.	95% CI	
					Lower limit	Upper limit
Symptoms PTSD EGSR	VSRSA	VSR	4.3736	0.182	−1.4416	10.1288
	VSRDA	VSR	12.2885^*^	0.000	5.5934	18.9835
	VSRDA	VSRSA	7.9449^*^	0.012	1.4308	14.4589
				
Stress DASS	VSRSA	VSR	1.7495	0.142	−0.4258	3.9248
	VSRDA	VSR	4.4919^*^	0.019	2.0106	6.9732
	VSRDA	VSRSA	2.7424^*^	0.000	0.3707	5.1141
				
DASS Anxiety	VSRSA	VSR	1.4766	0.182	−0.4911	3.4442
	VSRDA	VSR	4.6475^*^	0.000	2.3703	6.9247
	VSRDA	VSRSA	3.1709^*^	0.002	0.9760	5.3659
					
DASS Depression	VSRSA	VSR	1.5583	0.171	−0.4821	3.5988
	VSRDA	VSR	4.2875^*^	0.000	1.9136	6.6614
	VSRDA	VSRSA	2.7292^*^	0.015	0.4364	5.0220

*p < 0.05.

A hierarchical binomial logistic regression analysis was performed (Table 3) in which the predictor variables included those corresponding to the estimates of the different types of resources. The dichotomous variable *having been revictimized* (Yes/No) constituted the dependent variable. The resulting model was significant (*p* < 0.023) at a significance level of 0.05 and the goodness-of-fit test indicated that the model was able to explain 12.4% of the variance of the dependent variable (Nagelkerke’s corrected *R*^2^ = 0.124) when including all variables in the third step. In the first step, none of the material resources variables was significant. In steps 2 and 3, only social support was a significant predictor with an effect size of 0.97 (*p* < 0.021) and an inverse relationship with respect to membership in the group of revictimized women, such that greater social support corresponded to a lower risk of revictimization. None of the emotional resources introduced into the model in step 3 proved significant.

**Table 3:** Hierarchical binomial logistic regression.

	Step 1			Step 2			Step 3		
	*β*	*SE*	Exp(B) [CI].	*β*	*SE*	Exp(B)	*β*	*SE*	Exp(B) [CI].
Material Resources									
Income level	−0.195	0.127	0.823[0.64−1.06]	−0.134	0.132	0.875[0.68−1.13]	−0.101	0.138	0.904[0.69−1.19]
Employability^a^	−0.447	0.337	1.418[0.65−3.07]	0.360	0.401	1.433[0.65−3.15]	0.343	0.418	1.409[0.62−3.20]
Social resources									
Social support				−0.028^*^ [Table-fn tfn4]	0.011	0.972[0.95−0.99]	−0.028^*^ [Table-fn tfn4]	0.012	0.973[0.95−0.99]
Emotional resources									
Emotional regulation^b^							0.542	0.361	1.72[0.85−1.07]
Avoidance coping							0.069	0.043	1.071[0.99−1.17]
Self-negative coping							−0.059	0.041	0.943[0.87−1.02]
Positive coping							0.006	0.031	1.006[0.95−1.07]
Constant	0.893^*^	0.384	2.443	2.61^*^ [Table-fn tfn4]	0.811	13.621	1.42	1.22	4.132
*R^2 Nagelkerke^*			0.019			0.073			0.124
−*2 Log likehood*			222.645			215.748			208.838
ΔR*^2^*			0.019			0.054			0.060
									

a reference category is employed women.

b reference category is group with effective emotional regulation.

* *p <* 0.05.

After the binary logistic regression analysis, a hierarchical multinomial regression analysis was performed including the same predictor variables, but differentiated according to the type of revictimization suffered (same aggressor vs. different aggressors), to check whether the model improved and provided any new information by differentiating between the different types of revictimization (Tables 4, 5). The analysis showed that the model remained significant at a significance level of 0.01 (*p* < 0.005) and increased the percentage of variance explained to 19% (Nagelkerke’s corrected *R*^2^ = 0.187), an increase in variance of 50.8% over the previous model. Following the binary model procedure, current income, and the victim’s employability (Yes/No) were introduced in the first step as proxies for material resources but neither variable was found to be significant. In the second step, material resources were maintained and social support was introduced, which was positively related to membership in the group of VSR women, taking as a reference the VSRSA group. That is, with an effect size of 1.027 (*p* < 0.011), the VSR women tended to have greater social support than VSRDA women. Finally, in the third step, emotional resources were introduced, and significant results were found for emotional regulation variables and positive reappraisal coping strategies. Specifically, women assaulted on a single occasion were 2.93 times more likely to belong to the effective emotional regulation group than women revictimized by multiple aggressors (ExpB = 2.934; *p* < 0.026). Likewise, the odds of belonging to the group of women revictimized by multiple offenders increased by 16% for each point increase in positive reappraisal when compared to those women who had been assaulted on one occasion (ExpB = 0.863; *p* < 0.009) and by 12% when compared to women revictimized by the same offender (ExpB = 0.891; *p* < 0.028). In this third step, social support remained positively associated with belonging to the VSR group with respect to the VSRDA group (ExpB = 1.038; *p* < 0.008) and also with belonging to VSRs with respect to VSRSAs (ExpB = 0.974; *p* < 0.05). Material resources continued to have no relevant weight in the model.

**Table 4:** Hierarchical multinomial logistic regression using VSRDA as the reference group.

Type		Step 1			Step 2			Step 3		
		*β*	*SE*	Exp(B) [CI].	*β*	*SE*	Exp(B) [CI].	*β*	*SE*	Exp(B) [CI].
**VSR**	Material Resources									
	Income level	0.210	0.132	1.234[0.95–1.60]	0.140	0.138	1.150[0.878–1.507]	0.216	0.182	1.241[0.87–1.77]
	Employability^a^	0.164	0.400	1.178[0.54–2.58]	0.245	0.414	1.278[0.568–2.876]	0.277	0.530	1.319[0.47–3.72]
	Social resources									
	Social support				0.027^*^	0.010	1.027[1.01–1.05]	0.038^**^	0.014	1.038[1.01–1.07]
	Emotional resources									
	Emotional regulation^b^							1.076^*^	0.484	2.934[1.14–7.58]
	Avoidance coping							0.066	0.052	1.068[0.97–1.18]
	Self-negative coping							−0.003	0.041	0.997[0.92–1.08]
	Positive coping							−0.147^**^	0.057	0.863[0.772–0.964]
	Constant	0.181	0.487		−1.361	0.773		−1.959	1.421	
	*R^2 Nagelkerke^*			0.021			0.054			0.187
	*−2 Log likehood*			88.692			414.347			345.317
	Δ*R^2^*			0.021			0.033			0.133
										
**VSRSA**	Material Resources									
	Income level	0.150	0.137	1.161[0.89–1.52]	0.140	0.140	1.150[0.87–1.51]	0.168	0.170	1.183[0.85–1.65]
	Employability^a^	−0.283	0.419	0.754[0.33–1.71]	−0.195	0.428	0.823[−0.356–1.903]	−0.148	0.509	0.863[0.32–2.34]
	Social resources									
	Social support				0.010	0.010	1.010[−0.99–1.03]	0.013	0.012	1.013[0.99–1.04]
	Emotional resources									
	Emotional regulation^b^							0.773	0.467	2.166[0.87–5.41]
**VSRSA**	Self-negative coping							0.002	0.039	1.002[0.93–1.08]
	Avoidance coping							0.009	0.048	1.009[0.92–1.11]
	Positive coping							−0.115^*^	0.053	0.891[0.80–0.99]
										
	Constant	0.308	0.498		−0.280	0.732		0.514	1.256	
	*R^2 Nagelkerke^*			0.021			0.054			0.187
	*-2 Log likehood*			88.692			414.347			345.317
	ΔR*^2^*			0.021			0.033			0.133

** *p* < 0.01 and

* *p <* 0.05.

a reference category is women with employment.

b reference category is group with effective emotional regulation.

To interpret table correctly it is necessary to keep in mind that a negative sign on the regression coefficient B and an Exp(B) less than 1 indicate that the reference group (VSRDA) is more likely to have a high score on that predictor than the group to which it is being compared. VSRDA = women revictimized by different aggressors; VSRSA = women revictimized by the same aggressor; VSR = women victimized on one occasion; VSR = women victimized by the same aggressor; and VSR = women victimized on one occasion.

**Table 5:** Hierarchical multinomial logistic regression using VSR as reference group.

		Step 1			Step 2			Step 3		
Type		*β*	*SE*	Exp(B) [CI].	*β*	*SE*	Exp(B)	*β*	*SE*	Exp(B) [CI].
**VSRSA**	Material Resources									
	Income level	−0.060	0.102	0.941[0.77–1.15]	0.000	0.106	1.000[0.81–1.23]	−0.047	0.148	0.954[0.71–1.28]
	Employability^a^	−0.447	0.337	0.640[0.33–1.24]	−0.440	0.344	0.644[0.33–1.26]	−0.424	0.457	0.654[0.27–1.60]
	Social resources									
	Social support				−0.017^*^	0.009	0.983[0.966–1.00]	−0.025^*^	0.013	0.974[0.95–0.99]
	Emotional resources									
	Emotional regulation^b^							−0.304	0.392	0.738[0.34–1.59]
	Avoidance coping							−0.056	0.045	1.032[0.94–1.13]
	Self-negative coping							−0.005	0.034	1.005[0.94–1.07]
	Positive coping							0.032	0.046	1.032[0.94–1.13]
	Constant	0.126	0.396		1.081	0.678		2.472	1.214	
	*R^2 Nagelkerke^*			0.021			0.054			0.187
	*-2 Log likehood*			88.692			414.347			345.317
	ΔR*^2^*			0.021			0.033		7	0.133

To interpret table correctly it is necessary to keep in mind that a negative sign on the regression coefficient B and an Exp(B) less than 1 indicate that the reference group (VSR) is more likely to have a high score on that predictor than the group to which it is being compared. VSRDA = women revictimized by different aggressors; VSRSA = women revictimized by the same aggressor; VSR = women victimized on one occasion; VSR = women victimized by the same aggressor; and VSR = women victimized on one occasion.

a reference category is women with employment.

b reference category is group with low emotional regulation.

* *p <* 0.05.

## Discussion

Revictimization in women victims of Intimate Partner Violence is a serious problem that still has limited development in the scientific literature. Knowing more about the role played by the psychological and emotional factors of the victim herself in her revictimization is a perspective that, far from blaming the victim, can contribute to the exploration of which factors can moderate the impact of revictimization.

The present study aimed to study the weight of different social and emotional material resources in predicting being victimized on a single occasion (VSR), revictimized by the same aggressor (VSRSA) or being revictimized by different aggressors (VSRDA), with special interest in the analysis of emotional resources that have been largely ignored in research on revictimization.

Firstly, the results show the importance of taking into account the distinction between women victimized by a single or multiple aggressors when determining the clinical adjustment of the victims. Thus, the latter presented greater post-traumatic symptomatology, stress, depression, and anxiety than those women who were revictimized by the same partner, or who were victimized on a single occasion. Moreover, the greatest differences were found between the group of those not revictimized and those revictimized by multiple aggressors, which points to a dose/response effect according to the type of victimization suffered (Iverson et al., 2013). Differentiation between types of revictimization has also been shown to be an important moderator of the multinomial model posited. Only when distinguishing between the three groups of victims were emotional resources found to be significant in predicting revictimization. These results are in line with those found in Goodman et al. (2005), where, when differentiating exclusively between revictimized and non-victimized women, only social support and coping strategies were predictive. Thus, the type of revictimization, whether by the same or different aggressors, is confirmed as a factor that could improve the fit between the results of the LPI research and reality.

Secondly, material resources, particularly victims’ employability and income level, did not predict revictimization despite that which was found in previous studies (Caetano et al., 2005; Orke et al., 2018; Person, 2018). It is possible that the sample’s low variability in income level at least partially explains this result, as 70% of the women did not have an income which exceeded €1,000 per month.

Thirdly, our results indicate that social support functions as a protective factor against revictimization by significantly predicting membership in the group of non-revictimized women. The protective role of social support against revictimization has been repeatedly found in previous studies. The fact that non-revictimized women are those with greater social support is congruent with studies that relate the severity and duration of violence to distance from family and friends, as a consequence of perceiving their support as useless when women return to the violent relationship (Goodkind et al., 2003; Krenkel, 2014). From this point of view, the loss of social support would be one of the consequences of revictimization which, in turn, would increase the risk of suffering from it.

Fourthly, in relation to emotional resources, the results show that poor emotional regulation is a risk factor for revictimization by multiple aggressors. The importance of emotional regulation in the occurrence of emotional problems and recovery from traumatic events has recently been highlighted. Thus, a recent meta-analysis found that PTSD, regardless of the type of traumatic incident that generated it, it is characterized by a general dysregulation of emotions that acts as a precursor of clinical symptomatology (Seligowski et al., 2015). Taking into consideration, these findings and taking into account the worse adjustment of women revictimized by multiple aggressors in all dimensions of clinical symptomatology evaluated, the results found would indicate that worse emotional regulation could be favoring the continuity of revictimization through clinical symptomatology, especially when such revictimization occurs at the hands of multiple aggressors. This interpretation is compatible with that found by Muñoz-Rivas et al. (2021), where it was shown that the interaction effect between PTSD symptomatology and emotional regulation was significant exclusively in the group of women revictimized by multiple aggressors but not in other groups of victims. These results are consistent with the fact that being a victim of several episodes of IPV at the hands of different aggressors can be understood as a form of greater severity due to the greater associated psychopathology, the worse emotional regulation, and the lesser social support received with respect to women victims of a single episode of violence, or revictimized at the hands of the same aggressor. The importance of emotional regulation highlights the utility of examining which specific emotional regulation strategies are a key in the study of revictimization, as we have begun to do with strategies for coping with violence. In a first approach, it was found that women revictimized in IPV with poor emotional regulation and high PTSD symptomatology tend to use specific regulation strategies based on emotional impoverishment, which implies difficulty in experiencing emotions and poor emotional processing, and which involves negative mood and intrusive emotions (Muñoz-Rivas et al., 2021).

In addition to emotional regulation, another emotional resource that has generated an interesting result has been the coping strategy of positive reappraisal, which is more frequently used by women victims of different aggressors than by women not revictimized or revictimized by the same aggressor. Positive reappraisal has classically been considered an “engagement” strategy, associated with healthy stress coping patterns (Iverson et al., 2013). Therefore, initially this result is paradoxical, since in our study positive reappraisal is used more precisely by the group of women who have demonstrated the worst clinical adjustment and poor emotional regulation skills. Nevertheless, Zink et al. (2006) emphasized that reappraisal of the violent relationship plays a key role in learning to live with abuse, especially in young women. The interpretation of this result is clarified, first of all, from the framework of learned helplessness, which proposes that those women with more severe and prolonged histories of revictimization develop attitudes of resignation and hopelessness due to the absence of control over their circumstances (Walker, 1979), which makes it more challenging to end the abusive relationship (Di Basilio et al., 2022). Thus, according to the results found, faced with the impossibility of changing or escaping from their situation, women revictimized by multiple aggressors would resort to positive reappraisal to change their interpretation of their situation. Moreover, younger women are more likely to use this coping strategy in their situation of helplessness, as Zink et al. (2006) have already found. This explanation in terms of the learned helplessness theory is even more plausible if one takes into account that, in the current study, almost 40% of the women revictimized by different aggressors stated that they were likely to be assaulted again by other partners in the future when compared to 20% in the other two groups.

Secondly, another factor that could contribute to the greater use of reappraisal strategies in women who have been revictimized by multiple perpetrators is social support. Thus, in the review by Waldrop and Resick (2004), it has been suggested that battered women’s shelters may be a useful place where women can begin to stop reevaluating their situation and minimize its seriousness by hearing from other victims that abuse is negative. Similarly, Kacot and Goodman (2003) found that “engagement” coping strategies, far from favoring the adjustment of victims of IPV, were associated with greater post traumatic symptomatology, especially when the participants had little social support. Considering that our results show lower social support precisely in those women revictimized by multiple aggressors, it would be interesting to test two possibilities. Either the mediating role of social support in the use of positive reappraisal coping strategies, or, conversely, whether the use of positive reappraisal strategies could be acting as a barrier to seeking social support, taking into account that in the recent systematic review conducted by Robinson et al. (2021) it was found that precisely the lack of awareness of violence is the main barrier to seeking help in women victims of IPV.

In any case, these results support the utility of using an intra-individual approach in the study of coping strategies as opposed to trans-transituational models that assume the functionality or dysfunctionality of the strategies without taking into account the context in which they are used (Waldrop and Resick, 2004; Puente-Martinez et al., 2021). In this study, it has been found that under circumstances of violence maintained over time in different relationships, in women with low social support and younger age, the victims are more likely to use strategies of reevaluation of the situation to adapt to it in the short term, although this could mean maintaining the situation of violence. However, more studies are needed to specify under what conditions some coping strategies are more likely to be used than others. For instance, Han et al. (2022) found that women victims of physical injury and sexual abuse are more likely to use coping strategies based on the absence of reaction to violence, similar to learned helplessness.

### Limitations

This work has some limitations. Firstly, the results can only be generalized to battered women who have filed a police report against their partner, which should be evaluated as an indicator of severity in an IPV context. Secondly, the cross-sectional design does not enable us to examine temporal relationships between the variables, which prevents us from knowing whether the different resources play a role of antecedent or consequence with respect to the different types of revictimization. Thirdly, the variables “avoidance coping strategies” and “positive reappraisal” did not show a significantly linear relationship with the logit of the independent variable. However, the quadratic relationship was not significant either, so although a linear relationship could not be demonstrated, it could not be ruled out either. Finally, this study only includes a part of possible coping strategies, so it would be of interest to carry out research that contemplates a greater number of dimensions and their interaction with other variables such as social support, with which associations have already been identified in previous studies (Kacot and Goodman, 2003, Goodman et al., 2005). Other factors of interest, such as the type of violence suffered or its severity, have not been taken into account in this study neither. These characteristics of violence have been shown to play a moderating role in previous studies (Testa et al., 2003; Kuijpers et al., 2012; Di Basilio et al., 2022) so it would be advisable to evaluate them and include them in future analyses.

### Future clinical implications

The findings of this study have substantial clinical implications. This research points to the possibility of interventions with female IPV victims that focus on emotional resources. Given that cognitive behavioral therapy is less effective when there is intense affect dysregulation (Taylor and Harvey, 2010), victims may benefit from acquiring affect regulation skills (Ford et al., 2011), especially for women with a history of cumulative victimization. Another important contribution of this study points to discarding the usual recommendation of generic training in coping strategies or emotional regulation considered adaptive (Iverson et al., 2013; Puente-Martinez et al., 2021). According to the review by Waldrop and Resick (2004), non-specific training in problem-solving skills is not effective in the group of revictimized women when there are circumstances of lack of social support. Instead, it would make more sense to train women to begin to develop their own capacity to identify what role their coping strategies play in sustaining the violence, as well as to help them begin to build a support network. As Di Basilio et al. (2022) claim in their recent study, revictimization should be understood as a dynamic process involving multiple forces that push the victim to leave or continue the relationship. Interventions should move away from trying to follow standardized programs to try to help the victim identify which are the personal and contextual factors that promote and accelerate the process of disengagement with the perpetrator. In short, it is necessary to take into account the specific context of the victims when designing an intervention and, in particular, this work puts the focus on women who are more vulnerable due to their history of revictimization with multiple aggressors and low social support, who will tend to use strategies of re-evaluation of the situation in order to adapt to it in the short term, running the risk of maintaining their problem in the long term.

## Data availability statement

The data are not publicly available due to privacy and ethical restrictions. Data are available upon reasonable request and upon the signature of a confidentiality agreement from the research team. Requests to access the datasets should be directed to marina.munoz@uam.es.

## Ethics statement

The studies involving human participants were reviewed and approved by the Institutional Review Board of Universidad Autónoma de Madrid (CEI-941720). The patients/participants provided their written informed consent to participate in this study.

## Author contributions

AB, IM, and MI: conceptualization and supervision. AB and IM: methodology, formal analysis, data curation, and writing–review and editing. AB: resources and writing–original draft preparation. All authors contributed to the article and approved the submitted version.

## Funding

This work has been made possible by a research grant from the Ministry of Science, Innovation, and Universities of Spain (PGC2018-096130-B-I00).

## Conflict of interest

The authors declare that the research was conducted in the absence of any commercial or financial relationships that could be construed as a potential conflict of interest.

## Publisher’s note

All claims expressed in this article are solely those of the authors and do not necessarily represent those of their affiliated organizations, or those of the publisher, the editors and the reviewers. Any product that may be evaluated in this article, or claim that may be made by its manufacturer, is not guaranteed or endorsed by the publisher.
